# 2025 Acknowledgment of AAC Reviewers

**DOI:** 10.1128/aac.00334-26

**Published:** 2026-04-01

**Authors:** Cesar A. Arias

**Affiliations:** 1 Houston Methodist Hospital 23534 , Houston, Texas, USA; 2 Houston Methodist Research Institute 167626 , Houston, Texas, USA; 3 Weill Cornell Medical College 12295 , Houston, Texas, USA

## EDITORIAL

*Antimicrobial Agents and Chemotherapy*’s (AAC) high-quality and timely publication of groundbreaking scientific research are critically dependent on the outstanding efforts of the Editorial Board Members (https://journals.asm.org/journal/aac/board-editors) who generously contribute their time, wisdom, and talents to our journal. The editors of AAC also acknowledge the *ad hoc* referees who have contributed reviews in the past year, and we express our deep appreciation for their contributions to maintaining the journal’s scientific rigor, relevance of research, and high clinical impact.

Rob Aarnoutse

Lilian M. Abbo

Hazem F. M. Abdelaal

Kamilia Abdelraouf

Henrietta Abodakpi

Anson Abraham

Robert B. Abramovitch

Erwin Adams

Paul C. Adamson

Yau Adamu

David Addiss

Ahmed Adebowale Adedeji

Max W. Adelman

Nisheeth Agarwal

Surya D. Aggarwal

Manuj Ahuja

Jingwen Ai

Samuel L. Aitken

Onno W. Akkerman

Murat Akova

Ana Alastruey-Izquierdo

Kevin Alby

Alexander Maximilian Aldejohann

Jeffrey D. Alder

Katie J. Aldred

Sarah Alexander

Kevin Alexandre

Jan-Willem Alffenaar

Babukar Alhaji Kolo

Afrah Alkazemi

Lao-Tzu Allan-Blitz

Karel Allegaert

George P. Allen

Abdulaziz S. Alobaid

Adit B. Alreja

Mohammad H. Alshaer

Guzman Ignacio Alvarez

John Alverdy

Jorge Amich

David R. Andes

Andres Andrade Dominguez

Alberto Antonelli

Juan Manuel Anzola

Kotaro Aoki

Amir Arastehfar

Marcelo Araújo

Jorge Arca-Suárez

Gabriele Arcari

Sevtap Arikan-Akdagli

Samuel L. M. Arnold

Corinne Arpin

Cindy G. Arvidson

Yoann Augagneur

Yossef Av-Gay

Sean N. Avedissian

José Avendaño-Ortiz

Michael A. Bachman

Bhupesh Bagga

Bing Bai

Fernando Baquero

Aleksandra Barac

Daniel Barkan

Raul G. Barletta

Dan H. Barouch

Ivan Bassanini

Robert H. Bates

Thomas Bauch

Hannelore Bax

Inga Bazukyan

Sarah R. Beattie

Alejandro Beceiro

Karsten Becker

Ronen Ben-Ami

Tom Beneke

Agathe Béranger

Paul Beringer

Luiz E. Bermudez

Joseph S. Bertino

Dany Beste

Souvik Bhattacharyya

David Bhella

Adarsh Bhimraj

Gabriele Bianco

Tihana Bicanic

André Birgy

Ibrahim Bitar

Matthew G. Blango

Lucia Blasco

Lucas Boeck

Balazs Bogos

Joseph Bondy-Denomy

Remy A. Bonnin

Robert A. Bonomo

P. Brandon Bookstaver

Andrew M. Borman

Helena Ingrid Boshoff

Alberto Bosque

Germán Bou

Robert Bowden

Dana R. Bowers

Jeff M. Boyd

Patricia A. Bradford

Steven B. Bradfute

John S. Bradley

Axel A. Brakhage

Miriam Braunstein

Andrea Brenciani

James Brien

Scott D. Briggs

Maximo Brito

Rachel S. Britt

Michael S. M. Brouwer

Ashley N. Brown

Jochem Berend Buil

Jürgen B. Bulitta

Zackery Bulman

Elif Bulut

Charles Burdet

David Burger

Vincent Burrus

Karen Bush

Richard A. Calderone

Christian Callebaut

Emmanuelle Cambau

Francois Caméléna

Rafael Cantón Moreno

Valerio Capitani

Travis Carlson

Yehuda Carmeli

Glen P. Carter

Cecilia G. Carvalhaes

Yvan Caspar

Mariana Castanheira

Santiago Castillo-Ramírez

Dario Cattaneo

Vincent Cattoir

Casey Cazer

Laura K. Certain

John D. Chan

Liana C. Chan

Daniel B. Chastain

Som S. Chatterjee

Chengye Che

Jessica Chellappah

Liang Chen

Qingquan Chen

Yunyu Chen

Qin Cheng

Vesa Cheng

Kelly Chibale

Kathleen Chiotos

Jin Gun Cho

Suein Choi

Andrew Chou

Joseph Chow

Hin Chu

Daniela Maria Cirillo

Anthony J. Clarke

Daniel Lee Clemens

Javier Cobo

Ellen E. Codd

Sarah A. Coggins

Pier Giorgio Cojutti

Angela Colbers

Che C. Colpitts

Brian Conlon

Melissa D. Conrad

Teresa M. Coque

Oliver A. Cornely

Nicolas W. Cortes-Penfield

Richard Court

Robert A. Cramer

Tim R. Cressey

Fiona Cresswell

Dean C. Crick

Maria L. S. Cristiano

Delphine Croisier

Liwang Cui

Kyle W. Cunningham

Christina A. Cuomo

Michael Cynamon

Krystyna Dąbrowska

George L. Daikos

Renato A. DaMatta

Eric Dannaoui

Veronique A. Dartois

Alexandra Dassonville-Klimpt

J. Muse Davis

Noémie de Cacqueray

Cesar de la Fuente-Nunez

Josué de Moraes

Laura E. de Vries

Saskia N. de Wildt

Stefan L. Debbert

Jean-Winoc Decousser

Sarah Dellière

Jeroen den Hertog

Abhishek Deshpande

Aaron Sriram Devanathan

Guo Deyin

Neeraj Dhar

Gunaraj Dhungana

Jose A. Di Conza

Vincenzo Di Pilato

Andreas H. Diacon

Gill Diamond

Thomas Dick

Joseph P. Dillard

Nicholas A. Dillon

Genevieve Shirley Dobihal

Ulrich Dobrindt

Hana Maria Dobrovolny

Roberto Docampo

Fernando Docobo-Perez

Jean-Denis Docquier

J. Stone Doggett

Yohei Doi

Justin J. Donato

Hao Dong

Scott Jorge Dos Santos

Benoît Doublet

Thomas J. Dougherty

Khalid M. Dousa

Kevin J. Downes

Djamel Drider

Xiang-Dang Du

Paul M. Dunman

Lauren Dutcher

Raphaël E. Duval

Michael D. Edstein

Stephanie Lynn Egge

Khalid Eljaaly

Craig D. Ellermeier

Elodie Gautier-Veyret

Andrea Endimiani

Iuliana V. Ene

Hyungjin Eoh

Alice L. Erwin

German Esparza

Jose A. Este

Jaime Esteban

Benjamin Andrew Evans

David H. Evans

Marco Falcone

Séamus Fanning

Maha R. Farhat

Nikos Fatsis-Kavalopoulos

Paola Favuzza

Anthony R. Fehr

Sizhou Feng

Tabassum Ferdousi

Aude A. Ferran

Scott G. Filler

Natalie A. Finch

Jacqueline Findlay

L. Mark Fisher

Shawn Flanagan

Renee N. Fleeman

Courtney V. Fletcher

Steven L. Foley

Stephanie A. Fong

Jarrod R. Fortwendel

Majie C. Foster

Evelyn J. Franco

Karen M. Frank

Kristi L. Frank

Scott G. Franzblau

Andrew J. Fratoni

Daniel E. Freedberg

Jean-Marie Frère

Lena E. Friberg

Niels Frimodt-Møller

Frieder Fuchs

Kevin Fuller

Coralie Fumeaux

Lucy L. Furfaro

Fajri Gafar

Ana Cristina Gales

Danilo Cesar Galindo Bedor

Nan Gao

Gao Peng

Guillermo García-Effron

Kevin W. Garey

Sylvie Garneau-Tsodikova

Milo Gatti

Purushottam V. Gawande

Susanne Gebhard

Martin Gengenbacher

Giuseppe Gentile

Pallavi Ghosh

Daniele Roberto Giacobbe

Liezl Gibhard

Joy E. Gibson

Philip M. Giffard

Christian M. Gill

Jason J. Gill

Eva Gluenz

Youri Glupczynski

Marek Gniadkowski

Karla P. Godinez Macias

Joanna B. Goldberg

Gustavo H. Goldman

Rui Gong

Bruno Gonzalez-Zorn

Angel Gonzalez

Mark Gonzalez

Victor Gonzalez

Ana Gorgulho

Friedrich Götz

Sylvain Goutelle

David E. Greenberg

David Greenblatt

Matthieu Grégoire

Nicolas Gregoire

Mark S. Gresnigt

Fabio Gsaller

Jingmin Gu

Paul Gubbins

Severin Gudima

Carlota Gudiol

Jeremie Guedj

F. Peter Guengerich

François Guérin

Beatriz Guerra

John S. Gunn

Yinjuan Guo

Hubertus Haas

Adam Hafner

Ferry Hagen

Ghady Haidar

Morgan Hakki

Ruth M. Hall

Otavio Hallal Ferreira Raro

Hiroshi Hamamoto

Ahmed Mufeed Hamdi

Kamal Hamed

Margaret R. Hammerschlag

Jin-Hee Han

Lingxi Han

Kirsten K. Hanson

Sarah V. Harding

Dariusz Hareza

Christopher Harmer

Llinos Gwawr Harris

Vasili Hauryliuk

Jane Hawkey

Rod Hay

Aaron Hefferman

Eric L. Hegg

Kristin Hegstad

Emily L. Heil

Henry Heine

Jürgen Held

Georg Hempel

Andrew Hemphill

Isabelle G. Henshall

Marta Hernández-García

María Patricia Hernández-Mitre

Janet A. Hindler

Elizabeth B. Hirsch

Pak-Leung Ho

Carolyn L. Hodo

Tobias M. Hohl

Jennifer L. Hoover

John Horton

Paul A. Hoskisson

Zheng Hou

Yu-Ying Phoebe Hsieh

Alice J. Hsu

Bryan Hsu

Fupin Hu

Fenglei Huang

Szu-Wei Huang

Michael D. Huband

Anna R. Huppler

Julian G. Hurdle

James C. Hurley

Ashraf S. Ibrahim

Hiroaki Ihara

Christelle M. Ilboudo

Sébastien Imbert

Francesco Imperi

Takashi Inui

Yasushi Itoh

Ram Iyer

Paul J. Jackson

Michael R. Jacobs

William R. Jacobs

Jichan Jang

Nerea Jauregizar

Katy Jeannot

Jorgen Skov Jensen

Christian Jenul

Andrew Jezewski

Jhih-Hang Jiang

Kyung-Wook Jo

Adam Johnson

Jodie Johnston

Paul Johnston

Lavania Joseph

Julie Ann Justo

Chikara Kaito

Manjula Kalia

Deepika Kannan

Sara M. Karaba

James A. Karlowsky

Marios Karvouniaris

Airat R. Kayumov

Sara Keller

Allan Kengo

Ayesha Khan

Thilo Khöler

David Samuel Khoury

Nicolas Kieffer

Joong-Yub Kim

Si-Ho Kim

Paul R. Kinchington

Heather King

James E. Kirby

Kimberly A. Kline

Michele M. Klingbeil

Charlotte Kloft

Frank Kloprogge

Charles Knirsch

Kwan Soo Ko

Birgit Koch

Simon Erik Koele

James B. Konopka

Laura L. Kovanda

Robert Krause

Barry N. Kreiswirth

Laurent Kremer

Manju Y. Krishnan

Christopher J. Kristich

James W. Kronstad

Shih-Chi Ku

Christine J. Kubin

Vera Kühne

Ashlan J. Kunz Coyne

Daniel R. Kuritzkes

Sebastian G. Kurz

Oliver Kurzai

Joseph L. Kuti

Jason C. Kwong

Hanna Kadri Laas

Chih-Cheng Lai

Chih-Ho Lai

Gyanu Lamichhane

Iain L. Lamont

Frederic Lamoth

Cornelia Barbara Landersdorfer

Luce Landraud

Fanny Lanternier

Michael Lanzer

Paula Lapinskas

Roy Law

William S. Lawrence

Christophe Le Terrier

Minh Patrick Le

Nathan A. Ledeboer

Jean Y. H. Lee

Marcus C. S. Lee

Seung Hwan Lee

Todd C. Lee

Amy Legg

Philippe Lehours

Dario Lehoux

Stanley M. Lemon

Alexander J. Lepak

James Levin

James S. Lewis II

Russell Lewis

Feng Li

Guilian Li

Jian Li

Kewei Li

Ruichao Li

X. James Li

Xi Li

Xian-Zhi Li

Xuening Li

Yan Li

Yanpeng Li

Yijia Li

Chuan Poh Lim

Tze Peng Lim

Thomas Lin

Xiaorong Lin

Yi-Tsung Lin

Nilton Lincopan

Jeffrey Lipman

Samuel Lipworth

Dejun Liu

Po-Yu Liu

Qingyun Liu

Xin Yao Liu

Yanmei Liu

Catherine Llanes

Stephanie Lo

Shawn R. Lockhart

Thomas P. Lodise

Lisa Lombardi

Olga Lomovskaya

S. Wesley Long

Michael C. Lorenz

Arnold Louie

Nacer Lounis

Philip T. LoVerde

Fengmin Lu

Brigid Lucey

Zhao-Qing Luo

Joseph Lutgring

Luchao Lv

Michael A. Lyons

Khalid M. Dousa

Angela M. Early

Donna M. MacCallum

Conan MacDougall

Andrew Ryan Mack

Tokameh Mahmoudi

Thomas Maitre

Piotr Majewski

Anjali Majumdar

Nermina Malanovic

Lesibana Malinga

Prashant K. Mallick

Hedi Mammeri

Yukari C. Manabe

Alexander Mankin

Hilario Cuquetto Mantovani

Gabriel Davi Marena

Clotilde Marin

Miguel A. Martín-Acebes

Adela Martin-Vicente

Ronny Martin

Luis Martínez-Martínez

Luis R. Martinez

Murilo Martini

Stacey L. Martiniano

Anne-Grete Märtson

Ruth C. Massey

Alireza Matekolaee

Amy J. Mathers

Carina Matias

Yasuhiko Matsumoto

Yasufumi Matsumura

Elodie Matusik

Max Maurin

Patrick Mazi

Shonna M. McBride

Glenn Alan McConkey

Thomas H. McConville

Erin K. McCreary

Jennifer McDanel

Elizabeth McFarland

Patrick McGann

Andrew J. McLachlan

Robert McLaughlin

Sarah M. McLeod

Matthew B. McNeil

Khisimuzi Mdluli

Francis Megraud

Bernd Meibohm

Joseph Meletiadis

Fanfei Meng

Guy Meno-Tetang

Beatrice Mercorelli

Ella M. Meumann

Nneka Mezu

Malgorzata Mikulska

Darlene Miller

Jamie Miller

William R. Miller

Elizabeth Misas

Nagendra N. Mishra

Richa Misra

Satoshi Mitarai

Juan Mo

Maryam Moazeni

Satish Mocherla

Greg Moeck

Jennifer F. Moffat

Saswat Sourav Mohapatra

Jörg J. Möhrle

Maria F. Mojica

Wendy W. K. Mok

Israel Molina

Michelle Momany

Ian Robertson Monk

Ronaldo Morales Junior

Jacob Moran Gilad

James C. Morris

Naomi Morrissette

Gene D. Morse

Ikechukwu Benjamin Moses

Shakeel Mowlaboccus

W. Scott Moye-Rowley

Stephen Muhi

Gina K. Munier-Thomson

Jose M. Munita

Carol A. Munro

Daniel M. Musher

Lise Musset

Ahmed Nader

Soe Yu Naing

Theodore E. Nash

Roland Nau

Kelsie Nauta

Maribel Navarro

Jerry Nedelman

Ricardo Negroni

Daniel C. Nelson

Dionysios Neofytos

Chin Fen Neoh

Jeniel E. Nett

Olivier Neyrolles

Paul Nguewa

Robert A. Nicholas

David P. Nicolau

Kirsten Nielsen

Angela M. Nilius

Aaron Nilsen

Camus Nimmo

Lisa-Marie Nisbett

Tomoyasu Nishimura

David E. Nix

Gary J. Noel

Robert Norton

Angela Novais

Christian Nsanzabana

Eric L. Nuermberger

Dennis Nurjadi

Edward O'Brien

J. Nicholas O'Donnell

James P. O'Gara

Teresa R. O'Meara

Margaret Oates

Jenna Oberstaller

Lynette Isabella Ochola-Oyier

Kazutaka Oda

Audrey Odom John

Nick O'Donnell

Peter Oelschlaeger

Norio Ohmagari

Alessandra Oliva

Antonio Oliver

Eva Otoupalova

Michael Otto

Joel S. Owen

Belen P. Solans

Jerónimo Pachón

Malcolm G. P. Page

Rekha Pai Mangalore

Manjunath P. Pai

Lucia Pallecchi

Glen E. Palmer

Lucie Paloque

Navarat Panjasawatwong

Vincenzo Papa

Costas C. Papagiannitsis

Genovefa A. Papanicolaou

Krisztina M. Papp-Wallace

Paschalis Paranos

Tanya Parish

Joo Youn Park

Sally R. Partridge

Salina Parveen

Nimish Patel

David Paterson

Aditya S. Paul

Federico Pea

Richard Pearson

Qi Pei

Charles A Peloquin

Xuanxian Peng

José M. Pérez-Victoria

Federico Perez

Eva Pericolini

Brian M. Peters

Gilles Peytavin

David H. Peyton

Beatrix Pfanzagl

Rebecca Pinder

Jose Enrique Piñero

Johann Pitout

Joe Pogliano

Jason M. Pogue

Stefan H. Pöhlmann

Norbert Polacek

Yury S. Polikanov

Husain Poonawala

Aimee D. Potter

Sally-Ann Poulsen

Spyros Pournaras

Pablo Power

Roger K. Prichard

Michael J. Pucci

Rosana Puccia

Hong Qin

Susan Raber

Saki Raheem

Govindan Rajamohan

Stuart A. Ralph

Gauthan Ramakrishnan

Jayapradha Ramakrishnan

Bernardo Ramírez-Zavala

Elisa Ramos-Sevillano

Jolene Ramsey

Gauri G. Rao

Rino Rappuoli

Philip N. Rather

Kerstin K. Rauwolf

Binod Rayamajhee

Michael D. Reed

Adriana Renzoni

Gregory Resch

Fredrik Resman

Kelly Reveles

Jean-Louis Reymond

Nathaniel James Rhodes

Louis B. Rice

Volker Rickerts

Thomas V. Riley

Nicole Robbins

Jason A. Roberts

Gregory T. Robertson

Christine Robin

D. Ashley Robinson

Joana Rocha-Pereira

G. Marcela Rodriguez

Juan Bautista Rodriguez

Julieta Rodriguez

Keith A. Rodvold

Paul D. Roepe

P. David Rogers

Kyle H. Rohde

Adriana E. Rosato

Warren E. Rose

Gian Maria Rossolini

Coleman Rotstein

Christopher M. Rubino

Aileen Rubio

Etienne Ruppé

Elena Rustchenko

Jeffrey M. Rybak

Mustafa Sadek

Hassan Safi

George Sakoulas

Takaya Sakura

Max Salfinger

Sam Salman

Mirella Salvatore

John C. Samuelson

Gerardo Junior Sanchez Garcia

Dominique Sanglard

Maurizio Sanguinetti

Toyotaka Sato

Sarah W. Satola

Teiji Sawa

Luis M. Schang

Frieder Schaumburg

Marc H. Scheetz

Joshua T. Schiffer

Dirk Schnappinger

Erika Schor

Audrey N. Schuetz

Martin Schutten

Stefan Schwarz

Michael S. Seaman

W. Evan Secor

Stefan Seitz

Philip R. Selby

Armel Jackson Seukep

William M. Shafer

Theresa A. Shapiro

Umender Sharma

Samuel A. Shelburne

Wang-Huei Sheng

Norelle L. Sherry

Gauri Shirish Shetye

Da Shi

Ryan K. Shields

Eunjeong Shin

Min-Kyoung Shin

Thomas M. Shinnick

Neta Shlezinger

Bhavarth S. Shukla

Lynn L. Silver

Jared A. Silverman

Martin Šíma

Fekade Sime

Maria Simitsopoulou

Oriana Simonetti

Ashutosh Singh

Renu Singh

Vinayak Singh

Mahipal Sinnollareddy

Jean-Claude Sirard

Maryam Siroosi

Younes Smani

Nicholas M. Smith

Todd Smith

Hiie Soerg

Thierry Soldati

Yinggai Song

Alex Soriano

Cristina de Castro Spadari

James Spencer

John Spencer

Michele Spring

Michele D. Spring

Shashikant Srivastava

Nongluk Sriwilaijaroen

Henry M. Staines

Joseph F. Standing

Kenneth A. Stapleford

William J. Steinbach

Joerg Steinmann

Jannik Stemler

David Stepensky

Roger Stephan

Sophie Lena Stocker

Neil Stokes

Katharine E. Stott

Veronique Stove

Nicholas Streck

Sundharraman Subramanian

Gina Suh

Yao Sun

Sesh A. Sundararaman

Martin Sundqvist

Elin Svensson

Emma L. Sweeney

Sabira Tahseen

Masahiko Takahashi

Yasuhito Tanaka

Biao Tang

Lili Tao

Rick L. Tarleton

Joel Tarning

Pierre Tattevin

Dale Taylor

Tobias Tenenbaum

Christine B. Teng

Tanel Tenson

Rita Tewari

Abrar K. Thabit

Elizabeth J. Thompson

George Thompson

Michael Thy

Guo-Bao Tian

Giusy Tiseo

Marcelo Tolmasky

Andrew P. Tomaras

Steven Y. C. Tong

Arzu Topeli

Carlo Torti

Domenico Tortorella

Daan Touw

Mohamad-Ali Trad

Truc T. Tran

Ana Traven

Rihard Trebše

Sung-Pin Tseng

Thomas Tu

Nicholas A. Turner

Masaru Usui

Roya Vahedi-Shahandashti

Felice Valzano

Françoise Van Bambeke

Louvina Elizabeth van der Laan

Nicole N. van der Wel

Tjip S. van der Werf

Patrick Van Dijck

Welmoed van Loon

Wisse van Os

Willem Van Schaik

Brian C. VanderVen

Brian D. VanScoy

Shawn Vasoo

Juan Carlos Vázquez-Ucha

Luis Alberto Vega

Mario Venditti

Jonathan L. Vennerstrom

Michael P. Veve

Nicolas Veziris

Pierluigi Viale

Jorge E. Vidal

Xavier Vila-Farres

Alejandro J. Vila

Paolo Visca

Miguel Viveiros

Ivan Vokral

Cecilia F. Volk

Bao Gia Vu

Nikki J. Wagner

Catriona Waitt

Seth T. Walk

Jennifer N. Walker

Hui Wang

Jann-Yuan Wang

Jun Wang

Li Wang

Peigang Wang

Pengfei Wang

Qianlin Wang

Yang Wang

Yuhang Wang

Lynn Wardlow

Adilia Warris

Fumiya Watanabe

Richard J. Webby

Sharon Wein

Lukasz Weiner

Melanie Wellington

Theodore C. White

Nathan P. Wiederhold

Julia Willett

Dustin L. Williams

William Wilson

Marisa Winkler

William R. Wolowich

Rachel A. F. Wozniak

Gerard D. Wright

Yongning Wu

Yue-E Wu

Kelly L. Wyres

Meng Xiao

Jie Xing

Sifei Xing

Susan Xu

Zeling Xu

Mukesh Kumar Yadav

Pablo Yagupsky

Jason H. Yang

Katherine Yang

Soo-Jin Yang

Venkata Yellepeddi

Daniel K. Yeoh

Dongeun Yong

Yuanhai You

Chen Yu

Yunsong Yu

Jing Yuan

Yongyuth Yuthavong

Markus Zeitlinger

Francis Zekueng

Adrian M. Zelazny

Sheryl Zelenitsky

Lin Zeng

Helen I. Zgurskaya

Jing Zhang

Jiyu Zhang

Leiliang Zhang

Tianyu Zhang

Wenyan Zhang

Yan Zhang

Chunfu Zheng

Jiancang Zhou

Shuntai Zhou

Tieli Zhou

Xin Zhou

Yabin Zhou

Xiao Zhu

Xiangkai Zhuge

Oren Zimhony

Gang Zou

Jason E. Zucker

Simbarashe Peter Zvada

